# Piroxicam-β-Cyclodextrin: A GI Safer Piroxicam

**DOI:** 10.2174/09298673113209990115

**Published:** 2013-06

**Authors:** C Scarpignato

**Affiliations:** Clinical Pharmacology & Digestive Pathophysiology Unit, Department of Clinical & Experimental Medicine, University of Parma, Italy

**Keywords:** NSAIDs, piroxicam, piroxicam-β-cyclodextrin, coxibs, safety, GI risk, CV risk.

## Abstract

Although NSAIDs are very effective drugs, their use is associated with a broad spectrum of adverse reactions in the liver, kidney, cardiovascular (CV) system, skin and gut. Gastrointestinal (GI) side effects are the most common and constitute a wide clinical spectrum ranging from dyspepsia, heartburn and abdominal discomfort to more serious events such as peptic ulcer with life-threatening complications of bleeding and perforation. The appreciation that CV risk is also increased further complicates the choices of physicians prescribing anti-inflammatory therapy. Despite prevention strategies should be implemented in patients at risk, gastroprotection is often underused and adherence to treatment is generally poor. A more appealing approach would be therefore to develop drugs that are devoid of or have reduced GI toxicity. Gastro-duodenal mucosa possesses many defensive mechanisms and NSAIDs have a deleterious effect on most of them. This results in a mucosa less able to cope with even a reduced acid load. NSAIDs cause gastro-duodenal damage, by two main mechanisms: a physiochemical disruption of the gastric mucosal barrier and systemic inhibition of gastric mucosal protection, through inhibition of cyclooxygenase (COX, PG endoperoxide G/H synthase) activity of the GI mucosa. However, against a background of COX inhibition by anti-inflammatory doses of NSAIDs, their physicochemical properties, in particular their acidity, underlie the topical effect leading to short-term damage. It has been shown that esterification of acidic NSAIDs suppresses their gastrotoxicity without adversely affecting anti-inflammatory activity. Another way to develop NSAIDs with better GI tolerability is to complex these molecules with cyclodextrins (CDs), giving rise to so-called “inclusion complexes” that can have physical, chemical and biological properties very different from either those of the drug or the cyclodextrin. Complexation of NSAIDs with β-cyclodextrin potentially leads to a more rapid onset of action after oral administration and improved GI tolerability because of minimization of the drug gastric effects. One such drug, piroxicam-β-cyclodextrin (PBC), has been used in Europe for 25 years. Preclinical and clinical pharmacology of PBC do show that the β-cyclodextrin inclusion complex of piroxicam is better tolerated from the upper GI tract than free piroxicam, while retaining all the analgesic and anti-inflammatory properties of the parent compound. In addition, the drug is endowed with a quick absorption rate, which translates into a faster onset of analgesic activity, an effect confirmed in several clinical studies. An analysis of the available trials show that PBC has a GI safety profile, which is better than that displayed by uncomplexed piroxicam. Being an inclusion complex of piroxicam, whose CV safety has been pointed out by several observational studies, PBC should be viewed as a CV safe anti-inflmmatory compound and a GI safer alternative to piroxicam. As a consequence, it should be considered as a useful addition to our therapeutic armamentarium.

## INTRODUCTION

Although non-steroidal anti-inflammatory drugs (NSAIDs) are very effective, their use is associated with a broad spectrum of adverse reactions in the liver, kidney, cardiovascular system, skin and gut [1]. Gastrointestinal (GI) side effects are the most common and constitute a wide clinical spectrum ranging from dyspepsia, heartburn and abdominal discomfort to more serious events such as peptic ulcer with life-threatening complications of bleeding and perforation [2, 3]. The dilemma for the physician prescribing NSAIDs is, therefore, to maintain the anti-inflammatory and analgesic benefits while reducing or preventing their GI untoward effects.

The use of all medications increases with age and the elderly are at increased risk of the spectrum of adverse drug reactions. The occurrence of these complications depends on the presence (and number) of risk factors, and age is the most frequent and relevant of these factors. Thus patients at risk should be on prevention strategies including the use of the lowest effective dose of NSAID, co-therapy with a gastroprotective drug or the use of a cyclooxygenase-2 (COX-2) selective agent [4, 5]. Despite the best strategy to prevent lower GI complications has yet to be defined, treatment of associated *Helicobacter pylori* infection is also important when starting treatment with NSAIDs or aspirin, especially in the presence of an ulcer history [6, 7].

Unfortunately, however, gastroprotection is often underused and adherence to treatment is generally poor. Indeed, eleven observational studies in 911,000 NSAID users showed that 76% of the patients with at least one GI risk factor received no prescription for gastroprotective agents [8]. Furthermore, prescription of prophylactic gastroprotection adds to the pill burden in these patients and may complicate their daily regimens, leading to a non adherence rate exceeding 30% [9]. Therefore, in clinical practice few patients who need gastroprotection get it, and those who get it may not take it.

Although co-therapy with misoprostol or proton pump inhibitors (PPIs) is effective in preventing NSAID-induced gastro-duodenal damage [10, 11], a more appealing approach would be to develop drugs that are devoid of or have reduced GI toxicity. Currently, selective inhibitors of the inducible COX enzyme (often incorrectly referred to as coxibs^1^) offer the best chance for providing patients with an effective and safe anti-inflammatory therapy [13, 14]. Although several attempts (including enteric-coated or buffered preparations as well as the use of non acidic pro-drugs), have been disappointing [15], improved formulations, where conventional NSAIDs are complexed with phospholipids [16] or cyclodextrins [17], might have some chance of reduced topical irritancy.

During the last few years, great attention has been focused on cardiovascular (CV) adverse effects of COX-2 selective NSAIDs, which prompted to a re-evaluation of the CV (and global) safety profile of traditional (i.e. non-selective) compounds. The increased CV risk of COX-2 selective inhibitors has been well documented in RCTs and observational studies. Whereas this risk may be different according to dose, and patient baseline cardiovascular risk, more recent evidence points out that at least some, if not all, traditional NSAIDs may also increase that risk [18-20]. The renovascular effects of NSAIDs are also well known. Current evidence suggests that NSAIDs and coxibs have a similar incidence of these adverse effects, but with molecule-specific quantitative differences between the various drugs [21]. 

The Vioxx^®^ fallout [22] has created considerable public interest and increased the pressure on prescribers to find alternative medications displaying comparable anti-inflammatory activity and pain relief with acceptable CV, GI, and skin safety. After the withdrawal of COX-2 inhibitors, rofecoxib and valdecoxib, there was a significant decrease in coxib prescribing and a corresponding increase in non-selective NSAID prescriptions (with or without a proton pump inhibitor) [23, 24]. Despite the fact that coxibs and NSAIDs share the same CV and renal risks [21], physicians are looking at NSAIDs with renewed interest and need a re-evaluation of the risk/benefit ratio of these “old” drugs to make an appropriate choice.

A pharmacoutilization study in USA [23] found that - amongst the traditional NSAIDs - piroxicam ranked third, when the increase in monthly prescriptions per thousands patients after rofecoxib withdrawal was examined. Piroxicam is indeed a well-established NSAID, which has stood the test of time and used worldwide in the treatment of musculoskeletal diseases [25]. It belongs to the oxicam family of compounds [26] and displays a long half-life, allowing its once daily administration. A Cochrane review [27] has shown that piroxicam has an efficacy similar to that of other NSAIDs and of intramuscular morphine (10 mg), when used as a single oral dose in the treatment of moderate to severe postoperative pain, thus representing an alternative to other analgesics in various pain states. 

Although the tolerability of piroxicam is generally good and overlaps that of other NSAIDs, with gastrointestinal complaints being the most frequently reported adverse effects [28], evidence for the efficacy/safety profile of piroxicam has been repeatedly challenged since the early 1990’s. Notably, the Public Citizen’s Health Research Group (HRG) has petitioned three times the US Food and Drug Administration (FDA) to either remove the drug from the US market or restrict its use [29]. These petitions were subsequently denied by the FDA [30]. Piroxicam has also called the attention of the EMA [31] due to some observational studies, which suggested that - compared to other NSAIDs - it carries out a higher risk of adverse effects [32-38].

Several meta-analyses of observational studies [32, 33, 35, 39] have attempted to rank the relative GI adverse reactions attributable to various NSAIDs in the major European and US markets. Observational studies which reported high odd ratios (ORs) of GI bleeding for piroxicam had little precision with wide 95% C.I., whereas those reporting lower ORs had higher precision and were cohort studies with large sample sizes [40, 41]. Indeed, the design and quality of the studies appear to be strong independent predictors of the risk estimate; cohort studies were associated with lower risk estimates than case-control studies, and satisfactory studies were associated with lower risk estimates than unsatisfactory ones [40]. As a matter of fact, while quantitative syntheses of epidemiological studies suggest for piroxicam a harmful safety profile, indirect comparison of randomized clinical trials (RCTs) show similar to better safety [42]. 

To provide the “best evidence” [43] of piroxicam efficacy and safety, a meta-analysis, including 75 RCTs on 33,286 patients comparing this drug with other widely used NSAIDs, was performed [44]. The results highlighted a similar to better efficacy of piroxicam as compared to all other NSAIDs commonly prescribed in the management of musculoskeletal diseases. Along the same way, the overall and GI safety of piroxicam was also similar to (and sometimes better than) those of all other traditional NSAIDs.

This meta-analysis did not include CV events since no RCTs concerning the CV safety of piroxicam have been published. However, available observational studies [18, 45-47] have shown that, in clinical practice, piroxicam is not associated with a significant increase of the risk of either acute myocardial infarction or stroke. In addition, a meta-analysis of 54 studies dealing with blood pressure effect of non-selective NSAIDs [48] found that the increase in mean arterial pressure (after adjusting for amount of salt intake) was 3.59 mmHg for indomethacin, 3.74 mmHg for naproxen and only 0.49 mmHg for piroxicam, while decreasing (by 2.59 mmHg) after placebo. It is worth mentioning that blood pressure increasing effect of NSAIDs was evident solely in hypertensive subjects. Along the same lines, the piroxicam liver safety profile overlaps that of the other NSAIDs, being safer than etodolac, nimesulide and probably than diclofenac [49-52]. Finally, skin safety of piroxicam was also found similar to that of other commonly used NSAIDs [44]. A careful analysis of spontaneous reports from Italian regions [53] confirmed that the drug classes with the highest number of reports concerning *skin reactions *were antimicrobials followed by NSAIDs. Amongst them, aspirin and dipyrone use was associated with the highest reporting while piroxicam, naproxen and diclofenac were at the lowest level of the scale.

This “global” safety of piroxicam is mirrored by the persistence data. Persistence of treatment is likely to reflect the balance between efficacy and tolerability *as a whole*, since effective therapies with low toxicities are more likely to be continued than treatments with a less optimal efficacy/toxicity profile. Thus, discontinuation or switch rates are often a reliable marker of risk-benefit profile of medications. In a population-based study from MediCal [54], which included 15,343 cases and 61,370 controls, patients with arthritis tended to stay significantly longer on piroxicam compared to any of the other NSAIDs (namely diclofenac, naproxen and ibuprofen). These data are in line with a pharmacoutilization report [55], which describes the patterns of use of selective and non-selective NSAIDs from The Health Improvement Network (THIN) database in the UK and from the Pharmetrics database in the USA. The percentage of patients on continuous use of piroxicam at 31-60 days was larger than that of diclofenac, ibuprofen and naproxen, given at equivalent anti-inflammatory doses [55]. 

## TOWARDS A GI SAFER PIROXICAM

Gastro-duodenal mucosa possesses many defensive mechanisms and NSAIDs have a detrimental effect on most of them [56, 57]. This results in a mucosa less able to cope with even a reduced acid load. The presence of acid appears to be a *conditio* *sine qua non* for NSAID injury, which is indeed pH-dependent [58, 59]. Acid not only injures the mucosa, by H^+^ ion back diffusion from the lumen causing tissue acidosis, but also increases drug absorption, which is inversely proportional to drug ionization. NSAIDs cause gastro-duodenal damage by two main mechanisms Fig. (**1**): a physiochemical disruption of the gastric mucosal barrier and a systemic inhibition of gastric mucosal protection, through inhibition of cyclooxygenase (COX, PG endoperoxide G/H synthase) activity of the GI mucosa. A reduced synthesis of mucus and bicarbonate, an impairment of mucosal blood flow, an impaired epithelial cell turnover and an increase in acid secretion represent the main consequences of NSAID-induced PG deficiency [56, 57].

There is mounting evidence to suggest that gastric damage induced by non-selective NSAIDs does not occur because of COX-1 inhibition; rather, suppression of both COX-1 and COX-2 is necessary for damage [60-62]. However, against a background of COX inhibition by anti-inflammatory doses of NSAIDs, their physicochemical properties, in particular their acidity, underlie the topical effect leading to short-term damage [63]. Indeed, gastric injury (quantitated by Lanza score) correlated significantly with the pKa of the single compound: the lower the acidity of the drug, the less the mucosal damage.

Although less acidic than other widely used NSAIDs (like aspirin, diclofenac, naproxen and ibuprofen), piroxicam possesses anyhow a weakly acidic 4-hydroxy proton (pKa 5.1) while its selectivity towards COX isoenzymes is almost neutral, i.e. with a weak selectivity towards COX-1 Fig. (**2**) [64]. As a matter of fact, preventing the contact of free piroxicam with the gastric and duodenal mucosa *via* enteric coating reduces the short-term injury caused by the regular tablet formulation [65], thus supporting the importance of local effects. Rainsford [66] showed that esterification of acidic NSAIDs suppresses their gastrotoxicity without adversely affecting the anti-inflammatory activity. More recently, this avenue has been followed with the synthesis of new GI sparing compounds, where the NSAIDs are combined with nitric oxide (NO) or hydrogen sulphide (H_2_S) releasing moieties [67]. Another way to develop NSAIDs with better GI tolerability is to complex these molecules with cyclodextrins (CDs), giving rise to so-called “inclusion complexes” that can have physical, chemical and biological properties very different from either those of the drug or the cyclodextrin. Complexation of NSAIDs with β-cyclodextrin potentially leads to a more rapid onset of action after oral administration and improved GI tolerability because of minimization of the drug gastric effects [17]. One such drug, piroxicam-β-cyclodextrin (PBC), has been used in Europe for 25 years [68]. Such a successful approach has been applied to several other non-selective NSAIDs [69] and, more recently, to selective COX-2 inhibitors [70, 71].

The aim of the review is to summarize the pharmacology and clinical use of PBC pointing out its quicker onset of action as well as its better GI tolerability compared to piroxicam.

## CHEMISTRY OF PIROXICAM-β-CYCLODEXTRIN

The concept of host-guest chemistry has opened the way to the construction of supramolecular (inclusion) complexes with physicochemical properties superior to those of the guest molecule [72]. Naturally occurring examples of such complexes are heme and chlorophyll. Several types of host molecules have been synthesized, including crown ethers, cryptands, spherands, carcerands and CDs. All are able to act as *artificial receptors* and at least partially enclose guest molecules such as cations and drugs [73]. The concept of complementary host and guest molecules was introduced by 3 chemists (Charles Pedersen at Dupont Chemicals, Donald Cram at the University of California, Los Angeles, USA and Jean-Marie Lehn at the University of Strasbourg, France), who received the Nobel Prize in 1987 for their exceptional achievements [74].

In recent years, CDs have been recognized as an important group of pharmaceutical excipients enhancing drug solubility, dissolution, and bioavailability of poorly soluble drugs [75]. CDs are cyclic oligosaccharides of (α-1,4)-linked α-D-glucopyranose units. They are produced by enzymatic degradation of starch by a glucosyltransferase. The sugar units adapt a ^4^C_1 _chair conformation and orientate themselves in such a manner that the molecule forms a toroidal truncated cone structure Fig. (**3**). The free hydroxyl groups are situated on the outside of the ring, while the glycosidic oxygen bridges are situated in the inside, yielding a lipophilic inner cavity and a hydrophilic outer surface. The most common natural CDs are α-, β-, and γ-cyclodextrins, with 6, 7, and 8 glucopyranose units, respectively, with the optimal ring size being provided by β -CD [75]. 

These cyclic oligosaccharides are capable of forming non-covalent inclusion complexes with hydrophobic “guest” molecules, by including them inside their cavity. The molecules are trapped by non covalent intermolecular forces such as Van der Waals, hydrogen bonding and hydrophobic solvent forces [76]. Various types of drugs have been incorporated into CDs to take advantage of these properties, including corticosteroids, prostaglandins, antibacterials as well as NSAIDs [69]. Of the three different forms of CD, β-cyclodextrin has the most desirable dissociation characteristics for complexation with NSAIDs. It is particularly suited for complexation with lipophilic NSAIDs, because it has an internal hydrophobic milieu and the diameter of its inner cavity (0.76 nm) is the right size to accommodate an NSAID molecule [17].

CDs are chemically stable, water-soluble compounds that form complexes with water insoluble (lipophilic) molecules^2^. By this way they increase the aqueous solubility of poorly soluble drugs, thus increasing the availability of the drug at the site of absorption. When complexed with orally administered drugs, the primary use of cyclodextrins is to increase solubility, dissolution rate and stability of a given drug within the GI tract, decrease drug-mucosa contact time as well as increase GI absorption [78-80]. 

PBC is a 1:2.5 molecular complex of the oxicam-type NSAID, piroxicam, and the cyclic oligosaccharide, β-cyclodextrin Fig. (**3**). It contains the equivalent of 20 mg piroxicam in 191.2 mg of the complex molecule [81]. As a result of complexation, piroxicam looses its crystal structure; it is indeed an amorphous, hydrophilic, rapidly wettable compound, which dissolves rapidly. Using methods, such as differential scanning calorimetry, the freeze-dried PBC product has been confirmed to be a true inclusion complex rather than a dispersed mixture of the 2 separate components [82]. The crystal structure of the complex has been thoroughly investigated by X-ray diffraction analysis [83]. The results of this study unambiguously showed that β-CD is able to take up simultaneously two aromatic rings. The main driving forces for the complexation are provided *a)* by C-H…O interactions between the aromatic ring of the benzothiazinone scaffold in the guest and three glycosidic oxygen atoms at the inner surface of the β-CD and *b)* by hydrogen bonds involving the hydrophilic moiety of the guest as well as the primary and secondary ends of adjacent β-CD molecules Fig. (**4**).

Thanks to the poor solubility of piroxicam, the original preparation methods of the inclusion complex used organic solvents as media [84, 85]. However, their toxicities as well as the high concentration of residues in the final inclusion complex make these methods obsolete. The product can now be prepared by using supercritical carbon dioxide^3^ [86] without use of ammonia and any solvent. Supercritical carbon dioxide is devoid of any tocixity since, immediately after decompression, it returns to the gaseous state.

**Fig. (1):**
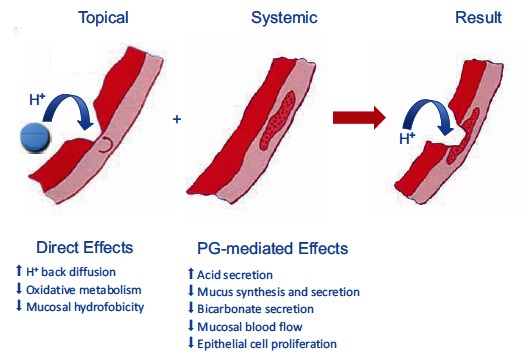
Main mechanisms underlying the upper GI toxicity of NSAIDs. The mucosal lesion (either erosion or ulcer) results from the combination of both topical (prostaglandin-independent) and systemic (prostaglandin-dependent) effects. COX inhibition by NSAIDs will give rise to diversion of arachidonate through the lipoxygenase (LO) pathway leading to enhanced leukotriene (LT) synthesis. These mediators cause vasoconstriction and release oxygen free radicals, which add to damage due to the impairment of mucosal defense.

In the complexed form, the aqueous solubility and dissolution rate of piroxicam are increased, leading to more rapid absorption from the upper GI tract and a shorter contact time with the gastric mucosa [81]. Therefore, it may be expected that complexation of piroxicam with β-cyclodextrin could protect the stomach and duodenum against *topical* GI damage. The systemic activity leading to inhibition of mucosal prostanoid synthesis and consequent impairment of mucosal defense mechanisms [3] (Fig. **1**) will not, of course, be modified by complexation.

The PBC complex dissociates in the gut Fig. (**5**). When administered orally to experimental animals, the intact carrier molecule (β-cyclodextrin) has negligible absorption, even at high doses [87], and no pharmacological effects [88, 89]. CDs are resistant to enzymes that hydrolyze starch, but bacteria in the colon are able to metabolize them to glucose and malto-oligosaccharides [88]. While dissolution rate and water solubility of PBC are enhanced at low pH (i.e. pH 2), thus promoting a quick absorption, at higher pH values (like those found in the intestinal lumen) its dissolution rate is somewhat below that of the uncomplexed drug [90]. This might limit the contact of uncomplexed piroxicam with the small bowel mucosa as well as its intestinal absorption, potentially reducing the occurrence of NSAID-enteropathy [91].

Cyclodextrins have intrinsically low toxicities, and are poorly absorbed from the GI tract following oral administration; therefore, safety concerns are minimal [17, 79, 89]. Safety studies of β-cyclodextrin conducted in animals have shown that it is virtually non-toxic and has no apparent carcinogenic potential [92-94]

Taking into account the physico-chemical properties of the β-cyclodextrin inclusion complex, the expected clinical benefits of PBC compared with uncomplexed piroxicam are:

- More rapid onset of the analgesic effect
- Improved topical upper GI tolerability.

The available experimental and clinical data do show that this is the case.

## PHARMACOKINETICS OF PIROXICAM-β-CYCLO DEXTRIN

The absorption of piroxicam from the β-cyclodextrin inclusion complex (PBC) is illustrated in Fig. (**5**). Because since piroxicam is only weakly associated with β-cyclodextrin (stability constant 90 mol^-1^·L), it is absorbed through the intestinal epithelium once the inclusion complex is in solution [81]. The β-cyclodextrin complex increases the absorption rate of piroxicam by increasing the dissolution rate of the drug. As the dissolved drug begins to penetrate the intestinal epithelium, the inclusion complex, acting as a reservoir, further dissociates to release more piroxicam. Following oral administration, β-cyclodextrin is minimally absorbed from the GI tract and the small absorbed fraction is essentially excreted in the urine without undergoing significant metabolism [81, 95]. Therefore, once piroxicam has been absorbed, β-cyclodextrin has no further effect on the NSAID pharmacokinetics.

Randomized crossover single- and multiple-dose studies of PBC in healthy volunteers have confirmed the expected more rapid absorption of this formulation, compared with uncomplexed piroxicam Fig. (**6**) [81, 96]. Other pharmacokinetic variables were similar between the two formulations. The faster absorption rate of PBC translates into a quicker onset of action (*see below*).

Piroxicam is a weak acid. As a consequence, absorption occurs primarily in the upper part of the small intestine and would therefore be dependent on gastric emptying rate [97, 98]. Studies have indeed shown that the presence of food increases the mean t_max_ of several oxicams, including piroxicam [99]. As expected, when PBC was administered after food the mean t_max_ occurred between 4.3 to 4.6 h, compared to 1.4 h in the fasting state [81]. Plasma levels of piroxicam, however, were higher than those measured after postprandial administration of the free drug [81].

Since β-cyclodextrin is only a carrier molecule that dissociates from piroxicam prior to absorption, PBC would be expected to display a post-absorption PK similar to that of free piroxicam. And indeed, available data confirm this assumption [96, 100]. 

The main route of elimination of piroxicam is metabolism, with only trace amounts (2 to 5%) of unchanged drug excreted in the urine. The principal metabolic pathway is hydroxylation to 5’-hydroxyl-piroxicam, which may be further conjugated to form the glucuronide. Piroxicam also undergoes cyclodehydration, the resulting metabolites of which are *N*-methylsaccharin and saccharin. There is some evidence that oxicams, including piroxicam, undergo enterohepatic recycling during elimination, i.e. they are excreted in bile and then reabsorbed in the small intestine. Piroxicam has a long t_1/2_β (40 to 63 hours), thus steady-state plasma concentrations are not achieved for approximately 7 to 14 days.

Pharmacokinetics of PBC was also evaluated in the elderly [101]. The mean plasma concentration of free piroxicam at the steady-state was significantly higher in elderly subjects (9.30±0.69 µg/ml) than in younger adults (6.24±0.58 µg/ml), a behavior similar to that of the uncomplexed drug [102, 103]. Both steady state plasma levels and areas under concentration-time curve (AUC) correlated significantly with age, suggesting dose reduction in the elderly.

A more recent study in patients with degenerative or inflammatory knee diseases [104] evaluated both plasma and synovial fluid concentrations of piroxicam after single administration of 20 mg of PBC. Piroxicam was strongly bound to serum albumin, with a free concentration of about 1%. However, this large protein binding did not appear to be a factor limiting the passage of piroxicam into synovial fluid. Indeed, the drug was detected right at the first aspiration, performed 30 min after dosing. The peak concentration of total piroxicam was reached later (after 6 h) and was lower in the joint compartment than in blood (1.31±0.76 µg/ml and 2.51±0.25 µg/m, respectively). In contrast, the mean t_1/2_ was much longer in the synovial fluid compared to the blood compartment (90.7 h *versus* 32.5 h). Finally, the synovial fluid/plasma ratio of the AUC was 0.39. These data show that piroxicam from PBC is absorbed soon enough to diffuse rapidly to the inflammed joints.

## FORMULATIONS OF PIROXICAM-β-CYCLODEXTRIN

Currently available formulations of PBC include regular tablets, sachets and a more recent effervescent formulation.

Compared to the tablet, piroxicam plasma levels after the sachet formulation are reached earlier [81]. The lag time needed for disintegration and dissolution of the solid formulation is indeed lacking when PBC sachet is given. In addition, gastric emptying of liquid dosage forms is faster than that of the solid ones [105, 106].

As expected, compared to the regular tablet, the effervescent formulation yielded a faster absorption rate, C_max_ being reached 15 min after oral administration. The two formulations were, however, bioequivalent [Acerbi, *personal communication*]. This kind of formulation displays - from a therapeutic standpoint - several advantages, the most relevant ones being [107, 108]:

### Fast Onset of Action

Effervescent tablets have the major advantage that the active compound is already in solution at the time it is taken. Thus, the absorption is usually faster and more complete than with conventional tablets. This is particularly helpful in treating acute symptoms like pain. Indeed, faster absorption translates into faster onset of action, a critical feature in the management of acute disabling symptoms.

### Accurate Dosing

Effervescent tablets enhance the absorption of a number of active ingredients compared to conventional formulations. This is because the carbon dioxide, created by the effervescent reaction, can induce enhanced active-ingredient permeability due to an alteration of the paracellular pathway. This pathway is the primary route of absorption for hydrophilic active ingredients, in which the solutes diffuse into the intercellular space between epithelial cells. It has been postulated that carbon dio- xide widens the intercellular space between cells, which leads to greater absorption of active ingredients (both hydrophobic and hydrophilic). The increased absorption of hydrophobic active ingredients could be due to the non-polar carbon dioxide gas molecule partition into the cell membrane, thus creating an increased hydrophobic environment, which would allow the hydrophobic active ingredients to be absorbed [109].

### Better GI Tolerability

Effervescent tablets dissolve fully in a buffered solution. Upper GI injury is often (albeit not always) pH-dependent: the lower the intragastric pH, the higher the mucosal damage [58, 59]. Buffered solution will quickly increase intragastric pH thus leading to mucosal protection. In addition, the quicker GI transit of the liquid formulation will reduce the contact time between the mucosa and the (potentially) noxious agent thereby reducing topical irritancy.

### Easy Swallowing

Since effervescent medications are administered in liquid form, they are easy to take as compared to tablets or capsules. The number of people who cannot swallow tablets or who dislike swallowing tablets and capsules is growing. Many diseased conditions require the patient or customer to swallow several tablets at a time. The elderly, in particular, have difficulty in swallowing tablets because of underlying swallowing [110] and/or esophageal motility [111] disorders.

## PRECLINICAL PHARMACOLOGY OF PIROXICAM-β-CYCLODEXTRIN

### Anti-inflammatory Activity

The anti-inflammatory activity of PBC was compared with that of the parent compound in different models of experimentally-induced inflammation in rodents. In carrageenin-induced pleurisy, treatment with either drug reduced both the exudate formation and leucocyte recruitment. The total leucocyte (both polymorphonuclear and mononuclear) count was however more strongly reduced by PBC [112]. These findings are in line with the results of Cadel & Bongrani [113], who showed that PBC reduced carrageenin-induced paw edema with an efficacy better than that of free piroxicam. The anti-inflammatory activity of both drugs was however similar in other models, like subcutaneous cotton pellet granuloma formation and complete Freund adjuvant-induced arthritis [112].

These data clearly show that inclusion of piroxicam with β-cyclodextrin does not reduce the anti-inflammatory effect of piroxicam, but rather can actually increase it, at least under some experimental conditions.

### Gastrosparing Activity

Preclinical studies also found that the gastric tolerability of PBC is better compared to that of free piroxicam [113]. Five hours after intragastric administration of the drug, the mucosal hemorrhagic area (both the length and surface) was significantly less (by 70-80%, p<0.05) with PBC compared to the parent compound [92]. Along the same lines, fecal blood loss was also significantly (p<0.03) lower [92].

The protective effects of β-cyclodextrin on gastric mucosa have recently been studied in an experimental model of cold stress-induced gastric ulceration [114]. Animals treated with piroxicam showed an ulcer index (14±1.8) significantly higher than that of control rats (3.8±0.4). However, when the β-cyclodextrin complex of piroxicam was given, gastric damage was actually lower (1.5±0.2) than that evoked by cold stress alone. Histological examination of the stomach of piroxicam-treated animals showed pronounced and marked ulceration with complete loss of the mucosa, extensive deposition of fibrin and dense neutrophil infiltration. On the contrary, animals treated with the β-cyclodextrin complex exhibited a normal gastric mucosa [114].

## CLINICAL PHARMACOLOGY OF PIROXICAM-β-CYCLODEXTRIN

Clinical pharmacological studies with PBC were mainly performed to confirm its analgesic and anti-inflammatory actions and investigate the onset of its pharmacologic activity in comparison with other NSAIDs, including free piroxicam.

### Analgesic Activity

Dental pain has been used as a model to evaluate analgesic efficacy since 1976 [115]. While a number of studies suggest that preoperative treatment with NSAIDs ensure good postoperative analgesia, the anatomical locations and molecular mechanisms underlying this pain killing effect was only recently studied. In a double-blind, randomized study Fornai *et al.* [116] assessed whether PG production at the surgical site accounts for the analgesia associated with the use of NSAIDs, given preoperatively to patients scheduled to undergo removal of an impacted third molar. They collected gingival specimens during tooth removal and after surgery and also evaluated patient’s subjective pain. It was found that pain intensity and PGE_2_ production, markedly increased after placebo, were significantly reduced at all time points by preoperative non-selective NSAIDs, like naproxen. After almost 30 years of use, dental pain has been accepted by FDA as a validated model for approval of new analgesic compounds [117]. In this connection, several clinical studies compared the analgesic activity of PBC to that of other non-selective NSAIDs in patients after removal of impacted third molar (*see clinical section*).

An alternative (non surgical) model that can be used in healthy volunteers is the evaluation of teeth nociceptive threshold to electrical stimulation [118]. In a double-blind, randomized, crossover study on 12 healthy volunteers [119] the nociceptive threshold of the six upper front teeth (left and right central and lateral incisors, and canines) was recorded every 15 min for 4 h after a single dose of PBC 20 mg, sodium naproxen 275 mg and sodium diclofenac 50 mg. The analgesic profile was similar for the three drugs. Significant effect was observed after 30 min, with the maximum effect reached between 45 and 60 min and lasting up to 150 min.

A comparative population pharmacokinetic-pharmaco dynamic analysis in patients with acute pain caused by musculoskeletical disorders [120] found that the onset of pain relief with PBC was faster than that observed with the uncomplexed piroxicam. Monte Carlo simulation showed that the time when at least 50% of the patients have a 75% probability of achieving meaningful pain relief (pain intensity difference ≥1) for PBC and parent compound (both at a dose of 20 mg) was about 0.5 and 1.5 hours, respectively.

### Gastrointestinal Tolerability

Gastric tolerability of PBC was investigated by using different techniques, either morphological (upper GI endoscopy and gastric mucosal scintigraphy) or functional (measurement of transmucosal potential difference and fecal blood loss).

#### Upper Gastrointestinal Tolerability: Endoscopy

Gastro-duodenal endoscopy represents the gold standard for assessing NSAID-induced GI damage, because it is a sensitive technique to assess NSAID-associated gastro-duodenal mucosal damage, but - performed in the usual way - it does not quantify the injury. To this end, following the common practice in experimental animal setting, some scores have been developed, the most widely used being the Lanza’s score [121]. Typically, the number of erosions and petechial hemorrhages are counted as endpoints, and a score is derived. Where the latter is done, a binary endpoint is usually presented (typically the proportion of subjects with Lanza grade ≥2, i.e. at least one erosion).

In a double blind, parallel group study [122] PBC or free piroxicam (both at the dose of 20 mg daily) were given for 14 days to healthy volunteers. Upper GI endoscopy was performed at baseline and after treatment and mucosal lesions scored according to Lanza *et al.* [121]. Results obtained are summarized in (Table **1**).

Similar results were obtained in an open study [123], where healthy subjects were randomly allocated to one of the following 4 treatments: group 1, PBC 20 mg daily; group 2, piroxicam 20 mg daily; group 3, indomethacin 100 mg daily; group 4, placebo. All the medications were given after breakfast for 14 consecutive days. Upper GI endoscopy was performed the last day of treatment. The gastric Lanza’s score for PBC (0.50±0.20) was significantly lower than that of piroxicam or indomethacin (2.06±0.50 and 2.25±0.50, respectively). Another double-blind study [124] evaluated gastro-duodenal mucosa before and after treatment with PBC (20 mg daily), piroxicam (20 mg daily) or placebo in 21 healthy volunteers. Four out of seven volunteers in the piroxicam-treated group withdrew because of severe GI symptoms and esophageal or gastro-duodenal lesions, while all the subjects treated with PBC or placebo completed the treatment. There was a significant (p<0.01) difference between endoscopic scores of piroxicam and placebo, but not between PBC and placebo. Although numerically distant (5.42±1.87 and 1.00±0.30), the difference between scores of piroxicam and PBC fell short of statistical significance, likely because of a β error. In the piroxicam group there were indeed 4/7 withdrawals.

While confirming that the stomach represents the major site of NSAID-associated upper GI injury [3], findings from these studies show that short-term treatment with PBC is less damaging than plain piroxicam (and indomethacin as well) to the gastric mucosa, substantiating results obtained from animal experiments. 

#### Upper Gastrointestinal Tolerability: Gastric Mucosal Scintigraphy

Sucralfate is a well-known mucosal protective compound, which displays both site- (i.e. mucosal coating) and cyto-protective activity on the entire GI mucosa [125]. Due to its affinity for eroded and ulcerated mucosa, it has been employed as an antiulcer compound in both animals and humans. Several investigations [*for review see* 126] demonstrated localized, prolonged adherence of labeled sucralfate to gastric and duodenal ulcers. Results of these studies suggested the use of radionuclide scanning techniques to diagnose peptic ulcer disease.

By using this non-invasive technique, 53 patients with osteoarthritis were given PBC (N=26) or piroxicam (N=27), both at 20 mg daily for 30 days, in a randomized fashion [127]. At the end of the treatment, 500 mg of ^99m^Tc-labeled sucralfate (185 MBq) were given by oral route, after an overnight fast, and anterior scintigraphic images collected at 30 min intervals for 2 h. The test was considered positive if all the 4 scintigrams revealed radioactivity uptake by gastric mucosa. Amongst patients taking PBC, 5 out of 26 (i.e. 19.2%) displayed a positive sucralfate scintigraphy while 10 out of 27 (i.e. 37.0%) piroxicam users had a positive test (p<0.01).

#### Upper Gastrointestinal Tolerability: Gastric Potential Difference

The luminal surface of the gastric mucosa is electrically negative when compared with the serosal one. There is a prominent lumen negative transmucosal potential difference in the stomach (gastric potential difference, GPD) [128]. GPD originates through an energy requiring process and has been shown to decline when oxygen availability is reduced [128]. Disruption of the gastric mucosal barrier by the so-called *barrier breakers* such as aspirin, ethanol and bile is associated with an increase in GPD [129], that is, a decrease in its negativity. Furthermore, a good correlation between the degree of histological damage and changes in GPD has been observed [128]. For these reasons, GPD has been used increasingly as an index of mucosal integrity [130].

By using a technique set-up in our laboratory, Santucci *et al.* [123] were able to show that GPD increase after single oral administration of PBC (20 mg) was significantly (p<0.001) lower than that observed with piroxicam (20 mg) or indomethacin (100 mg). Although the PBC-induced changes in GPD were larger than those observed after placebo, the difference fell short of statistical significance. These data do suggest that the topical gastric irritancy of PBC is minimal, if any, and are in line with the results of short-term endoscopic studies [63].

#### Gastrointestinal Blood Loss

It is common to find patients on treatment with NSAIDs who are anemic and this has commonly been attributed mistakenly to the anemia associated with chronic disease. These patients usually show a typical microcytic, iron deficient picture with a reduced hematocrit. A recent systematic review [131] showed that, at baseline, or with placebo, fecal blood loss amounts to 1 ml/day or below. With low-dose aspirin and some NSAIDs, average values may be two to four times this; anti-inflammatory doses of aspirin result in much higher average losses. A small proportion of individuals respond to aspirin or NSAIDs with much higher fecal blood loss of more than 5-10 ml/day. Fecal blood loss can be accurately determined by the ^51^Cr labeled red cells and, albeit not practical in the clinical setting, this technique has often been employed to evaluate GI tolerability of selective and non-selective NSAIDs [131]. The method involves stool collection for a number of days after injection of autologous erythrocytes labeled with ^51^Cr [132]. 

In a double-blind, double-dummy, parallel group study [124], healthy volunteers were randomly given either PBC (20 mg daily), piroxicam (20 mg daily) or placebo for 29 days. Whole stool samples were collected from 3 days before to the end of treatment, homogenized at the end of the study and counted in a large volume gamma counter. The mean fecal blood loss in PBC- and placebo-treated subjects remained within the normal range (≤ 2 ml/day) throughout the entire study period. On the contrary, bleeding increased significantly (over 9 times the basal value, p<0.005) in the piroxicam treated group.

Less consistent results were observed in the study by Warrington *et al.* [133]. In this trial similar cumulative blood losses were observed for PBC and piroxicam throughout 30 days of therapy, but an abrupt increase of bleeding thereafter occurred only with piroxicam. These investigators used a technique to quantitate blood loss, which was slightly different from the previous one. Methodological problems, notably collection of all stool samples, avoidance of interfering behaviors and suitable methods for measuring radioactivity in blood and stool can make a *reliable *measurement difficult and should be standardized. Differences in methodology can therefore explain discrepancies in findings.

## CLINICAL EFFICACY OF PBC VERSUS PIROXICAM AND OTHER NSAIDs

### Rheumatic Diseases and Other Musculoskeletal Disorders

Pain is a common reason for patients to visit their family physician [134, 135] and the numbers seeking treatment for pain is anticipated to rise as the population ages and chronic conditions such as osteoarthritis increase. In the UK, annually, more than 17 million prescriptions are written for anti-inflammatory and analgesic drugs [136]. Musculoskeletal pain is common and disabling, especially in the elderly. Since the conditions causing rheumatic pain, including osteoarthritis, inflammatory arthritis and soft-tissue conditions (such as tendonitis and bursitis), are, for the most part, not curable, pain control is paramount in order to maintain quality of life. Pain management should be multimodal, tailored to the individual patient, and will likely include a combination of both non pharmacological and pharmacological interventions. The widely used classes of drugs, namely simple analgesics (i.e. paracetamol), NSAIDs, stronger analgesics (i.e. opioids) and adjuvant drugs, each have unique and particular concerns regarding their adverse effect profiles. Since inadequate pain relief or dissatisfaction with a given treatment is a source of frustration and suffering for patients with chronic/persistent pain, an effective, safe and long-acting analgesic compound would be desirable. Indeed, balanced against the adverse effects of pain management medications, there is a need to be mindful of the widespread, often serious, adverse consequences of poorly managed pain itself.

Professional organizations including the American College of Rheumatology [137], American Pain Society [138] and European League Against Rheumatism [139] have published treatment guidelines within the past years to assist clinicians in achieving effective pain management. Safety is a core concern in all these guidelines, especially for chronic conditions such as osteoarthritis that require long-term treatment. Hence, there is a consensus among recommendations that paracetamol (acetaminophen) should be the first-line analgesic agent due to its favorable side effect and safety profile, despite a meta-analysis [140] showed that it is less effective in pain relief than anti-inflammatory drugs. 

Besides being a less powerful analgesic, paracetamol is not that safe either from a GI and CV perspectives, not to mention the well-known hepatotoxicity (especially at doses higher than 4 g daily) [141]. Indeed, a nested case control study [142] found that use of this compound is associated with a small but significant risk of upper GI complications (RR 1.3; 95% C.I. 1.1-1.5). The RR was 3.6 (95% C.I., 2.6-5.1) among paracetamol users of more than 2 g daily. In addition, while women from the Nurses’ Health Study, who reported occasional use of paracetamol, did not experience a significant increase in the risk of CV events, those who frequently (6-14 tablets/week) consumed it had a RR of 1.35 (95% C.I., 1.14 to 1.59) [143]. Finally, frequent paracetamol use is associated with an increased risk of hypertension both in women [144] and men [145]. The above findings are not surprising in the light of the recent discovery that paracetamol is indeed a selective COX-2 inhibitor in man [146].

A summary of randomized, controlled trials comparing PBC with other NSAIDs in patients with rheumatic diseases and other musculoskeletal disorders is presented in (Table **2**).

In these studies, PBC 20 mg daily was consistently found to be as effective as comparator NSAIDs, including uncomplexed piroxicam (20 mg daily), tenoxicam (20 mg daily), diclofenac (100 mg daily) and nabumetone (1000 mg daily), in relieving pain over periods of 2 to 12 weeks. In three studies that assessed analgesic activity in the first 24 hours, PBC had a more rapid onset of effect and produced more marked analgesia compared with comparator NSAIDs [147-149]. Furthermore, this piroxicam formulation was better tolerated than comparator NSAIDs in two trials [150, 151].

In the largest study, PBC was compared with uncomplexed piroxicam in 203 patients over 12 weeks. Both treatments significantly (p<0.05) reduced pain from baseline, but there were no significant differences between them in terms of analgesic efficacy [150]. Another study on 60 patients demonstrated that oral PBC had an onset, extent and duration of analgesic activity similar to that of intramuscular (IM) diclofenac or ketoprofen, but had greater global analgesic activity than diclofenac [152]. In a comparison with nabumetone, PBC was significantly more effective in the treatment of joint swelling after 2 weeks (p<0.05) and 4 weeks (p<0.01) of treatment, and was also significantly more effective for spontaneous pain, pain with passive movements and functional limitation after 4 weeks (p<0.05 for all) [148]. A longer-term study conducted in 107 patients also demonstrated that PBC had comparable efficacy to diclofenac after 6 months of treatment [153].

A recent paper [154] compared the clinical effects of PBC sachet to that of piroxicam tablet in patients with chronic low back pain. The patients in sachet group showed greater improvement in pain score and disability index than those who took piroxicam tablets. There were significantly (p< 0.05) lower sway velocity and intensity at almost all different conditions than baseline profiles in both groups.

### Primary Dysmenorrhea

Dysmenorrhea is the most common gynecologic complaint among adolescent females. Dysmenorrhea in adolescents is usually primary and is associated with normal ovulatory cycles and no pelvic pathology. In approximately 10% of adolescents with severe dysmenorrheic symptoms, pelvic abnormalities such as endometriosis or uterine anomalies may be found [155, 156]. Effective therapies include analgesic compounds, oral contraceptives and pharmacologic suppression of menstrual cycles. 

Since prostaglandins and leukotrienes play an important role in generating the symptoms of dysmenorrhea, NSAIDs are the most common pharmacologic treatment for this condition [157]. A recent Cochrane review including 73 RCTs [158] showed that NSAIDs are significantly more effective for pain relief than placebo (OR 4.50, 95% CI 3.85, 5.27) and paracetamol (OR 1.90, 95% CI 1.05 to 3.44). When NSAIDs were compared with each other there was little evidence of the superiority of any individual NSAID for either pain-relief or safety. However the available evidence had little power to detect such differences, as most individual comparisons were based on very few small trials [157]. Thanks to its quicker onset of analgesic activity as well as its long duration of action, PBC could be particularly suitable in this clinical setting.

Studies that compared the efficacy of PBC with other NSAIDs and/or placebo for the treatment of primary dysmenorrhea are shown in (Table **3**). One study conducted on 26 patients demonstrated that oral PBC was significantly more effective than placebo in decreasing pain and associated complaints, while rectal PBC was significantly more effective than naproxen sodium (p<0.05) [159]. In two multicenter, crossover studies, oral PBC 20 mg or 40 mg given once daily was significantly more effective than placebo and as effective as naproxen sodium and ibuprofen in relieving abdominal pain in 93 women with primary dysmenorrhoea. As in patients with musculoskeletal disorders [120], the onset of analgesic effect was more than two times faster with PBC compared to uncomplexed piroxicam. The duration of analgesia with PBC was also significantly longer than that observed with ibuprofen or placebo [160].

### Postoperative and Dental Pain

Acute postoperative pain differs from chronic pain because it is more transitory and affected by anxiety about the outcome of the surgical condition and often concern for suboptimal analgesia. Unfortunately, poorly controlled and persistent pain occurs after surgery and can be severe, which might increase the risk of a chronic pain state [161]. And indeed, for patients awaiting surgery, the possibility of severe acute postoperative pain is a major concern [162]. Uncontrolled postoperative pain can lead to delayed recovery from surgery, pulmonary dysfunction and hypoxia, and restriction of mobility with subsequent increased risk of thromboembolism. On the contrary, effective pain management improves patient satisfaction, decreases hospital stay, and shortens recovery of the postsurgical patient [161, 163].

Surgical tissue trauma results in the release of a large number of inflammatory mediators, including prostanoids. These mediators affect nociceptors, altering their firing threshold and sometimes causing direct stimulation. COX-2 plays a key role in the central neurological response to inflammation [164]. As a consequence, COX-2 inhibition with selective or non-selective compounds, which both cross blood-brain barrier, is effective for postoperative pain relief.

Patients undergoing minor surgery can be adequately managed with oral analgesics, such as NSAIDs, tramadol, and/or oxycodone. Those undergoing more extensive surgery usually require parenteral opioids or local analgesic techniques (regional block), sometimes in combination. In order to minimize adverse effects (such as sedation, respiratory depression, nausea and vomiting), the requirement for parenteral opioids could be reduced *via* co-administration of NSAIDs, which are indeed opioid-sparing [165].

(Table **4**) summarises studies investigating the analgesic efficacy of PBC compared with other NSAIDs in patients with postoperative pain or dental pain.

In three studies of patients with postoperative pain following orthopaedic surgery, the drug had similar analgesic efficacy to intramuscular (IM) uncomplexed piroxicam and tenoxicam [166-168]. On average, PBC had a significantly greater duration of analgesic effect than IM tenoxicam (9.6 *versus* 7.8 hours; p<0.01) [167]. The use of rescue analgesics was not statistically different between groups, but the time interval between test drug and the need for additional medication was 4.75±0.74 h and 6.92±0.82 h for PBC and IM piroxicam, respectively [168].

Studies conducted in patients with dental pain have also demonstrated the efficacy of PBC. In 298 patients who underwent third molar extraction, 30 minutes after tooth removal, the drug (20 mg) had a similar analgesic effect to paracetamol (500 mg) and a better analgesic effect than uncomplexed piroxicam (20 mg). However, after 4 hours, PBC and uncomplexed piroxicam had similar analgesic efficacy, which was significantly better than that of paracetamol [169]. In contrast, a comparison of meclofenamate sodium and PBC in 20 patients with periodontitis reported that meclofenamate sodium provided significantly better pain relief than PBC after 30 minutes (p<0.002), which however showed a longer duration of analgesic activity, being significantly more effective after 6 hours (p<0.03) [170].

The efficacy of PBC in a multimodal, balanced, perioperative analgesia was studied by Lauretti *et al.* [171]. They premedicated with either placebo or PBC (20 mg preoperatively) 48 patients scheduled for minor abdominal procedures. After intravenous administration of tramadol 1.5 mg/kg, anesthesia was induced with an intravenous loading dose of propofol and maintained with an intravenous infusion of the same drug (6-12 mg/kg/h) plus either saline or tramadol (at 1.2 mg/kg/h), atracurium, and a 2:1 nitrous oxide-oxygen mixture. In this clinical setting, the combination of tramadol and PBC provided better perioperative analgesia than tramadol alone.

### Other Pain States

Studies in patients with recurrent primary headaches, back pain and acute sport injuries have provided further evidence of PBC analgesic efficacy in a wide range of pain states. 

In a crossover study of 30 patients with primary headaches, treatment with PBC sachets led to a rapid decrease in pain intensity within 1 hour, and the effect over a 3-hour period tended to be greater than naproxen sachets [172]. Similarly, in patients with low back pain, PBC 20 mg once daily had a more rapid and more marked analgesic effect than etodolac 200 mg twice daily, and the duration of pain relief was generally more prolonged. At the end of treatment (day 7), painful symptoms had disappeared in more than 70% of PBC recipients, compared with 40% of patients treated with etodolac [173]. Among athletes with acute sport injuries, PBC led to a significantly greater reduction in pain from the second day onwards, compared with naproxen sodium (p<0.01) [174]. In a randomised study of 49 patients with acute ligament strains, PBC had a significantly greater analgesic effect than tenoxicam during the first 6 hours, with an overlapping efficacy thereafter [175].

## TOLERABILITY OF PIROXICAM-β-CYCLODEX TRIN *VERSUS* PIROXICAM AND OTHER NSAIDs

Piroxicam is the active moiety of PBC; hence, the adverse effects of the inclusion complex are, in general, those of piroxicam. However, because piroxicam has greater aqueous solubility and dissolution rate in the complexed form, and therefore more rapid absorption and shorter contact time with the gastric mucosa, the inclusion complex generally provides improved short-term gastric tolerability.

### Overall Incidence of Adverse Events in Clinical Trials

Total adverse event rates from an unpublished pooled analysis are presented in (Table **5**) [176]. This analysis included 13,559 patients from 42 published studies of PBC in patients with acute or chronic pain. The total incidence of adverse events in these studies was 9% with PBC, 20% with uncomplexed piroxicam and 25% with other reference agents.

A randomised, double-blind clinical trial conducted in 203 patients reported that PBC was associated with a lower incidence and severity of adverse events, compared with uncomplexed piroxicam [151]. Other trials that compared PBC with uncomplexed piroxicam [148], diclofenac [150, 152], and ketoprofen [152] concluded that PBC and the comparators were all well tolerated, with overall low incidence of adverse events. In a study comparing PBC and diclofenac, however the total incidence of adverse events was similar (44.2% vs 50.9%, respectively) [153]. 

### Gastrointestinal Adverse Events

The most frequent adverse events with PBC are GI events. A number of studies, both preclinical and clinical, suggest that PBC may have better GI tolerability than uncomplexed piroxicam and some other NSAIDs.

#### Clinical Studies

We have recently performed a detailed analysis of nearly 100 published and unpublished studies of PBC documenting the incidence of minor and major GI adverse events in a total of 29,190 patients. The incidences of minor GI events from 46 studies of PBC and comparator drugs used in the acute treatment setting are summarized in (Table **6**). The incidence of individual minor GI events was 0.07-1.37% for PBC, compared with 0-6.4% for piroxicam, 0.1-3.45% for other reference agents and 0.11-3.21% for placebo. Major and minor GI event rates were also analysed from 28 studies in the chronic treatment setting. Individual minor GI event rates ranged from 0.33-2.21% with PBC, compared with 1.73-11.92% for piroxicam and 0-6.05% for other agents (Table **7**). Major GI events were rare during chronic treatment with PBC (Table **8**). The incidence of major bleeding was 0.09% for PBC, 0% for piroxicam and 0.64% for other agents.

In a larger trial of 203 patients, GI tolerability was better with PBC than with uncomplexed piroxicam. Epigastric pain and pyrosis were the most common adverse events and occurred in 7.6% and 5.7%, respectively, of PBC recipients compared with 11.2% and 6.1%, respectively, of uncomplexed piroxicam recipients [151]. A 6 month, multicenter trial showed that PBC and slow-release diclofenac had similar overall GI tolerability. However, three patients in the PBC group withdrew because of GI adverse events, compared with nine in the diclofenac group [153]. In another smaller study comparing PBC and nabumetone, both treatments were well tolerated, but nabumetone had a higher incidence of GI adverse effects than PBC [149].

#### Other Adverse Events

Because the pharmacokinetics of piroxicam and PBC are similar (except for absorption), the most common non-GI adverse effects of PBC are expected to be similar to those observed with piroxicam (hypersensitivity reactions, headache, dizziness, vertigo, hearing disturbances and hematuria) [68]. Therefore, it is likely that the risk of cardiovascular, hepatic and renal adverse events with PBC is similar to the risk of these adverse events with piroxicam. 

Rarely reported adverse reactions from the PBC prescribing information [177] include edema, central nervous system reactions, dermal hypersensitivity, hypersensitivity reactions, renal function reactions, hematological reactions, changes in liver function parameters and hepatic reactions, palpitations and dyspnea [176].

## DISCUSSION

NSAIDs are an essential part of the therapeutic armamentarium despite their well characterized GI and CV risk profiles. Our increasing appreciation of these relationships and our new knowledge should allow a more safe and effective use of this class of drugs [3].

Physicians should not prescribe NSAIDs before taking a careful history and doing a physical examination so they have the information they need to balance the risks and benefits for individual patients. When GI and/or CV risk factors are present, appropriate preventive strategies (i.e. PPI use or low-dose aspirin) should be implemented from the very beginning and compliance to treatment be assessed regularly [178, 179], especially in the elderly [9]. Finally the appropriateness of NSAID prescription should be emphasized, i.e. to control inflammation and pain, rather than to control pain alone [180]. Only then can we hope to limit the expanding NSAID epidemic.

In this difficult scenario the choice of the NSAID is a challenging issue. Although selective COX-2 inhibitors have been synthesized with the aim to provide clinicians with a GI safe class of anti-inflammatory drugs [8, 181, 182], their safety has not always been confirmed. Indeed, despite a significant reduction in upper GI complications in the general population, the presence of more than one GI risk factors (age in addition to previous complicated peptic ulcer or co-morbidities and associated co-therapies) does impair their GI safety [183] and, often, gastroprotection with PPIs must be implemented in order to control dyspeptic symptoms [184, 185] and to prevent gastro-duodenal ulcers [186]. Although with molecule-specific quantitative differences between the various drugs, the CV [18-20] and reno-vascular [21] risk of selective COX-2 inhibitors represent an additional concern, which makes the evaluation of risk/benefit ratio of these compounds difficult.

The withdrawal of some COX-2 inhibitors, namely rofecoxib and valdecoxib, from the market contributed to raise anxiety and concerns about this class of drugs but also prompted a re-evaluation of the benefits (which are undisputed) and the risks (often forgotten in everyday clinical practice) of all selective and non-selective NSAIDs. Some old compounds (namely naproxen, ibuprofen and diclofenac) took advantage of being used as comparator drugs in the large clinical trials performed with celecoxib, rofecoxib and the other selective agents. As a consequence, a large database is today available for these traditional NSAIDs and the large amount of data (originating also from observational studies) allowed a better knowledge of the GI and CV risks associated with the use of these drugs. Being the CV risk an issue with both ibuprofen [187] and diclofenac [188] (and selective COX-2 inhibitors as well), naproxen has been recommended by experts [4, 189] and the American Heart Association [190] as the NSAID of choice in patients with CV risk factors. In those with concomitant GI risk, gastroprotection is always appropriate [4].

The NSAID class includes a large number of compounds, the chemical structure of which is very heterogeneous [191] and whose utilization pattern varies according to local availability, established use, reimbursement status and risk/benefit perception. All these drugs have been registered long time ago, when efficacy and safety requirements from the Regulatory Authorities were less stringent. As a consequence, only small (often not randomized and double-blind) trials, in which the safety assessment was seldom appropriate, are available for the majority of traditional NSAIDs.

The large meta-analysis on piroxicam efficacy and safety [44] included 75 RCTs on 33,286 patients and was performed with the aim of bridging the existing knowledge gap in the field. The analysis did not include CV events since no RCTs concerning the CV safety of piroxicam have been published. However, piroxicam was found to be safer - from a GI perspective - than naproxen, ibuprofen and diclofenac, amongst others Fig. (**7**). Since available observational studies [18, 45-47] have shown that piroxicam, like naproxen, is not associated with a significant increase in the risk of either acute myocardial infarction or stroke, this NSAID could be considered a suitable alternative to naproxen. It is conceivable that its CV safety be related to its long-lasting antiplatelet activity [192-195], which recalls that of naproxen [196-199].

Since the CV toxicity of selective and non-selective NSAIDs seems not to be preventable, even by concomitant administration of low-dose aspirin [19, 200, 201], selecting a CV safe anti-inflammatory drug should be the primary aim. On the contrary, upper GI damage associated with NSAID use could be reduced (but not avoided) by concomitant gastroprotection [4, 5]. The combination of piroxicam with a proton pump inhibitor or misoprostol might therefore provide a safe anti-inflammatory therapy from both CV and GI points of view. In this connection, PBC can offer a GI safer alternative to piroxicam.

Preclinical and clinical pharmacology of PBC do show that the β-cyclodextrin inclusion complex of piroxicam is better tolerated from the upper GI tract than free piroxicam, while retaining all the analgesic and anti-inflammatory properties of the parent compound. In addition, the drug is endowed with a quick absorption rate, which translates into a faster onset of analgesic activity, an effect confirmed in several clinical studies. 

The trials analyzed in the present review all show that PBC has a GI safety profile, which is better than that displayed by uncomplexed piroxicam. Along the same lines, an unpublished meta-analysis of Grayson (quoted by Müller & Simon [122]) on some 2000 patients, who were treated with PBC or free piroxicam, showed that, with the inclusion complex, there was a significantly lower incidence of GI side effects compared to that observed with the uncomplexed NSAID (8.5% *versus* 16.7%). The rate of GI adverse events with PBC was similar to that seen in patients given placebo (i.e. 7.2%).

After 25 years of use in Europe and South America, also PBC - like piroxicam - has stood the test of time and, on the grounds of its efficacy and safety, should be considered as a useful addition to our therapeutic armamentarium.

## Figures and Tables

**Fig. (2):**
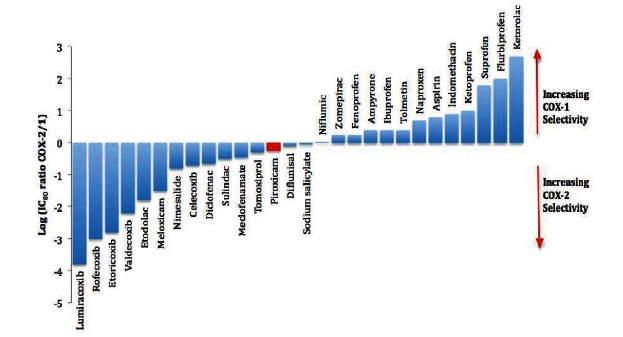
Relative selectivity of agents as inhibitors of human COX-1 and COX-2 dis- played as the ratio of IC_80_ concentrations. Inhibitor curves for compounds against COX-1 and COX-2 were constructed in a human modified whole blood assay and used to calculate IC_80 _concentrations. The IC_80_ ratios are expressed logarithmically so that 0 represents the line of unity, i.e., compounds on this line are equiactive against COX-1 and COX-2. Compounds appearing above the line are COX- 1-selective, those below the line COX-2- selective (from Warner & Mitchell [64]).

**Fig. (3):**
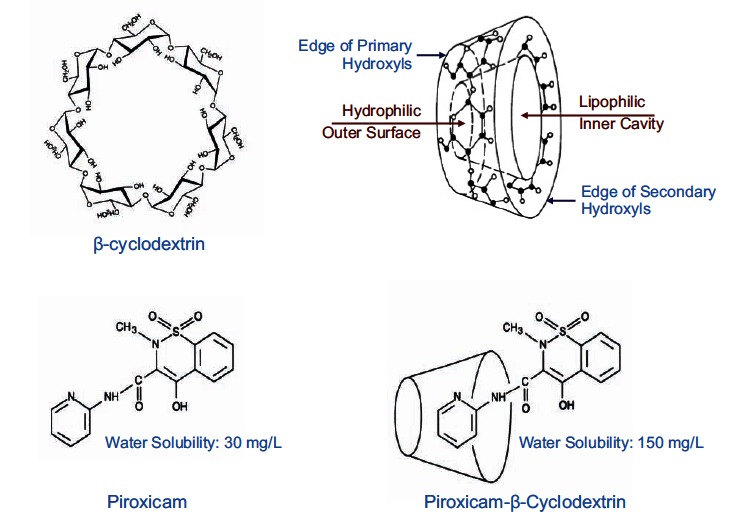
*On the top:* Structural formula of β-cyclodextrin together with its 3D structure, showing the lipophilic inner cavity and the hydrophilic outer surface. *On the botton:* Structural formula of free piroxicam and piroxicam-β-cyclodextrin.

**Fig. (4):**
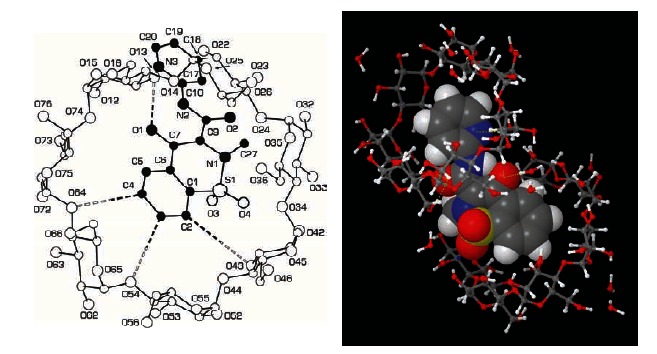
Crystallographic investigations on piroxicam-β-cyclodextrin. *On the left side:* the SCHAKAL drawing showing the host-guest interaction between piroxicam and β-cyclodextrin (from Chiesi-Villa et al., [83]). On the right side: the MAESTRO drawing showing the same host-guest interaction (courtesy of Dr. Andrea Rizzi, Computational Chemistry, Chiesi Farmaceutici S.p.A, Parma Italy). Both drawings have been generated with proprietary software packages (available at the following URL addresses: http://www.krist.unifreiburg.de/ki/Mitarbeiter/Keller/schakal.html and http://www.schrodinger.com)

**Fig. (5):**
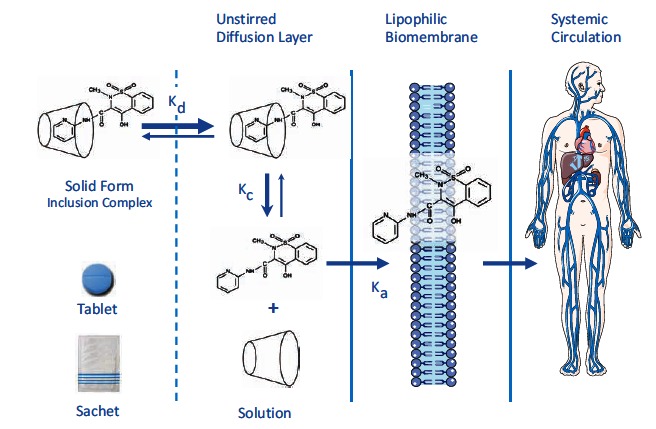
Dissolution and absorption of piroxicam from piroxicam-β-cyclodextrin. k_d _= dissolution constant k_c _= equilibrium constant for the formation of the inclusion complex k_a _= absorption rate constant.

**Fig. (6):**
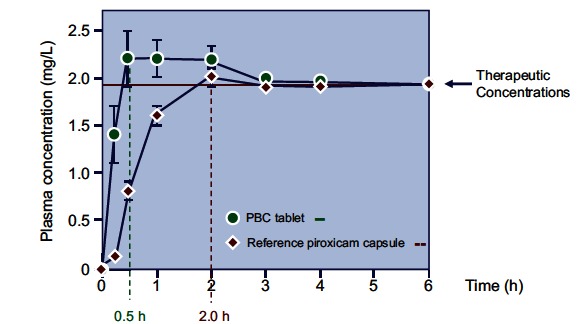
Mean piroxicam plasma levels after oral administration of PBC or free piroxicam in healthy volunteers. One PBC tablet (20 mg) or piroxicam tablet (20 mg) were given with 200 ml of water in a crossover fashion. Subjects were fasted but received a standard breakfast 2 h after drug administration (from Acerbi et al., [96]).

**Fig. (7):**
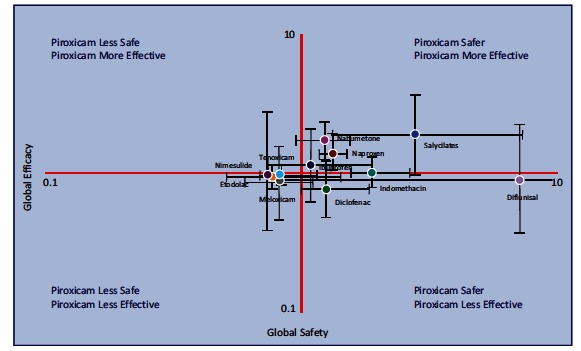
Summary findings regarding the relative efficacy and safety of piroxicam against other non selective NSAIDs. X (Global Safety) and Y (Global Efficacy) axes (logarithmic scales), displayed in red represent piroxicam, considered as the reference drug. Compared to naproxen and ibuprofen, the drug was found to display better safety and efficacy whereas, compared to diclofenac, piroxicam displayed a better safety and a (slightly) lower efficacy (from Richy *et al.* [44]).

**Table 1.** Changes in Endoscopic Lanza’s Score for the Stomach, The Duodenum and Both Sites in Healthy Male Volunteers Given PBC or Piroxicam (Both at 20mg Daily for 14 Days (From Müller & Simon [122])

Lanza’s Score*	Treatment		p value
	PBC	Piroxicam	
Total	2±4	5±4	0.03
Stomach	1±3	3±3	0.03
Duodenum	1±4	3±5	0.11

* Score values after 14 days *minus* score values at baseline.

**Table 2.** Summary of Randomised Controlled Trials Comparing the Analgesic Efficacy and Tolerability of PBC *versus* Other NSAIDs in Patients with Rheumatic Diseases and Other Musculoskeletal Disorders

Reference	Indication	Study Design	Duration	Drug/dosage (route)	No. of Patients	Analgesic Efficacy	Tolerability

Manzini *et al. *[147]	OA	R; SB; PG	7-15 days	PBC: 20mg od (oral)	20	PBC > Pir in first 24h (quicker onset of action); thereafter, PBC = Pir	Both treatments well tolerated
				Pir: 20mg od (oral)	20		

La Montagna *et al. *[148]	OA	R; PG	4 weeks	PBC: 20mg od (oral)	20	PBC > Nab in first 24h	Both treatments well tolerated
				Nab: 1000mg od (oral)	20		
						PBC > Nab for joint swelling at wk 2 and 4 and other pain parameters at wk 4	

Riccieri *et al. *[149]	OA	R; SB; PG	4 weeks	PBC: 20mg od (oral)	20	PBC > Dic in first 24h; PBC = Dic at wk 1, 2 and 4	Both treatments well tolerated
				Dic (SR): 100mg od (oral)	20		

Ambanelli *et al. *[150]	OA and RA	R; DB; PG	12 weeks	PBC: 20mg od (oral)	105	PBC = Pir	PBC better tolerated than Pir (lower incidence and severity of AEs)
				Pir: 20mg od (oral)	98		

Bonardelli *et al. *[151]	OA	R; SB; PG	8 weeks	PBC: 20mg od (oral)	15	PBC > Ten for pain on passive movement at wk 4.	PBC caused less GI mucosal damage (evaluated at endoscopy) than Ten
				Ten: 20mg od (oral)	15	PBC = Ten for all other parameters	

Tamburro *et al. *[152]	Acute musculoskeletal and articular pain	R; SB; PG	12 hours	PBC: 20mg (oral)	20	PBC = Dic and Ket in onset, extent and duration of pain relief	All three treatments well tolerated
				Dic: 75mg (IM)	20	PBC > Dic for global analgesic effect	
				Ket: 100mg (IM)	20		

La Montagna *et al. *[153]	OA	R; PG; MC	6 months	PBC: 20mg od (oral)	52	PBC = Dic at 6 months	PBC = Dic (total AE rates: 44.2% vs 50.9%)
				Dic (SR): 100mg od (oral)	55		

Abbreviations: AE=adverse event; DB=double-blind; Dic=diclofenac/diclofenac sodium; IM=intramuscular; Ket=ketoprofen; MC=multicentre; Nab=nabumetone; OA=osteoarthritis; od=once-daily; PBC=piroxicam-β-cyclodextrin; PG=parallel-group; Pir=piroxicam (uncomplexed); R=randomised; SB=single-blind; SR=sustained-release; Ten=tenoxicam; >indicates more effective than; =indicates as effective as.

**Table 3.** Summary of Randomised Controlled Trials Comparing the Analgesic Efficacy of PBC *versus* Other NSAIDs or Placebo in Patients with Primary Dysmenorrheal

					Efficacy Outcomes		

Reference	Study Design	Duration	Drug/dosage (route)	No. of Patients	Reduction in Pain Intensity	Duration of Pain Relief	Reduction of Associated Symptoms^a^

Costa *et al. *[159]	R; DB; PG	3.5 days (range, 2-6)	PBC: 20mg od (oral)	6	PBC oral > Plc	PBC oral = Plc	Day 2: PBC rectal > PBC oral > Plc > Nap
			PBC: 20mg od (rectal)	7	PBC rectal > Nap rectal	PBC rectal > Nap rectal	
			Nap: 550mg od (rectal)	7			
			Plc (oral)	6			

Dawood *et al. *[160] *Study 1*	R; DB; CO; MC	3 days	PBC: 20mg od (oral)	93	PBC 40mg = PBC 20mg = Nap > Plc (30 mins - 24 h)	PBC 40mg = PBC 20mg = Nap > Plc^b^ (6h)	
			PBC: 40mg od (oral)				
			Nap: 550mg + 275mg up to q6h (oral)				
			Plc (oral)				

Dawood *et al. *[160] *Study 2*	R; DB; CO; MC	3 days	PBC: 20mg od (oral)	93	PBC 40mg = PBC 20mg = Ibu > Plc (60 mins - 24 h)	PBC 40mg = PBC 20mg > Ibu = Plc^b^ (8h)	
			PBC: 40mg od (oral)				
			Ibu: 400mg up to q6h (oral)				
			Plc (oral)				

a Associated symptoms include headache, nausea and intestinal disturbances

b Determined by the proportion of patients who re-medicated. Abbreviations: CO=crossover; DB=double-blind; Ibu=ibuprofen; MC=multicentre; Nap=naproxen sodium; od=once-daily; PBC=piroxicam-β-cyclodextrin; PG=parallel-group; Plc=placebo; q6h=every 6 hours; R=randomised; >indicates more effective than; =indicates as effective as.

**Table 4.** Summary of Randomised, Controlled Clinical Trials Comparing the Analgesic Efficacy of PBC *versus* Other NSAIDs in Patients with Postoperative Pain or Dental Pain

Reference	Indication	Study Design	Duration	Drug/dosage (route)	No. of Patients	Efficacy Outcomes		

						Reduction in Pain Intensity	Duration of Pain Relief	Other Outcomes

Michelacci *et al. *[166]	Orthopedic surgery	R; DB; PG	24 hours	PBC: 20mg (oral)	12	PBC = Pir		Patient self-evaluation: PBC = Pir
				Pir: 20mg (IM)	12			

Simone & Oliani [167]	Orthopedic surgery	R; DB; PG	12 hours	PBC: 20mg (oral)	25	PBC = Ten up to 4h	PBC > Ten	
				Ten: 20mg (IM)	24	PBC > Ten from 6-12h		

Martens [168]	Orthopedic surgery	R; DB; PG	4 days	PBC: 20mg od (oral)	26	PBC = Pir	Pir > PBC^a^	Use of rescue medication: PBC = Pir
				Pir: 20mg od (IM)	24			

Dolci *et al. *[169]	Postextraction	R; DB; PG	4 hours	PBC: 20mg (oral)	74	At 0.5h: PBC = Prct > Pir = Plc		Patient self-evaluation: PBC = Pir > Prct > Plc
				Pir: 20mg (oral)	76	At 4h: PBC = Pir > Prct > Plc		
				Prct: 500mg (oral)	72			
				Plc (oral)	76			

Marcucci *et al. *[170]	Acute periodontitis	R; PG	6 hours	PBC: 20mg (oral)	10	At 0.5h: Mec > PBC		Patient self-evaluation: Mec > PBC
				Mec: 100mg (oral)	10	At 6h: PBC > Mec		

a As determined by the mean interval between study medication and requirement for additional medication on day 1. Abbreviations: db = double-blind; IM = intramuscular; Mec = meclofenamate sodium; od = once-daily; PBC = piroxicam-β-cyclodextrin; pg = parallel-group; Pir = piroxicam (uncomplexed); Plc = placebo; Prct = paracetamol; r = randomised; Ten = tenoxicam; > indicates more effective than; = indicates as effective as.

**Table 5.** Total Adverse Event Rates in a Pooled Analysis of Data From 42 Published Studies with PBC (Data On File, Chiesi Farmaceutici)

Type of Studies	No. of Patients^a^	Percentages of Patients with Adverse Events			
		Piroxicam-β-cyclodextrin^b^ (N=12 778)	Piroxicam (N=157)	Other^c^ (N=453)	Placebo (N=196)
Acute pain	8281	8%	13%	18%	6%
Chronic pain	5278	10%	27%	32%	NA
Total	13 559	9%	20%	25%	6%

a Patients in crossover studies are counted only once

b Includes all dosage forms and all doses

c Other reference agents in acute pain studies included tenoxicam, naproxen sodium, ibuprofen arginine, indomethacin, ketoprofen and tiaprofenic acid. Other reference agents in chronic pain studies included tenoxicam, diclofenac/diclofenac sodium, meclofenamate sodium, nabumetone, etodolac, ketorolac tromethamine and droxicam.

**Table 6.** Summary of Minor GI Events with PBC, Piroxicam, Other Reference Agents and Placebo in 46 Acute Treatment Studies From a Pooled Safety Analysis (Data on File, Chiesi Farmaceutici)

	Piroxicam-β-cyclodextrin^a^		Piroxicam		Other^b^		Placebo	
	N=10 292		N=203		N=956		N=904	
	N	%	N	%	N	%	N	%
Abdominal pain	87	0.85	1	0.49	5	0.52	1	0.11
Constipation	7	0.07	0	0.00	1	0.10	2	0.22
Diarrhea	34	0.33	0	0.00	6	0.63	7	0.77
Gastritis	38	0.37	8	3.94	33	3.45	4	0.44
Nausea	141	1.37	2	0.99	22	2.30	29	3.21
Vomiting	27	0.26	0	0.00	9	0.94	13	1.44

a Includes different dosage forms and different doses of PBC

b Other reference agents included uncomplexed piroxicam, tenoxicam, naproxen sodium, ibuprofen arginine, indomethacin, ketoprofen and tiaprofenic acid.

**Table 7.** Summary of Minor GI Events with PBC, Piroxicam and Other Reference Agents in 28 Chronic Treatment Studies From a Pooled Safety Analysis (Data On File, Chiesi Farmaceutici)

	Piroxicam-β-cyclodextrin^a^		Piroxicam		Other^b^	
	N=15 040		N=579		N=314	
	N	%	N	%	N	%
Abdominal pain	50	0.33	34	5.87	3	0.96
Constipation	50	0.33	19	3.28	0	0.00
Diarrhea	159	1.06	25	4.32	5	1.59
Dyspepsia	85	0.57	69	11.92	17	5.41
Gastritis	50	0.33	11	1.90	19	6.05
Nausea	333	2.21	31	5.35	8	2.55
Vomiting	53	0.35	10	1.73	0	0.00

a Includes all dosage forms and all doses of PBC

b Other reference agents included tenoxicam, diclofenac/diclofenac sodium, meclofenamate sodium, nabumetone, etodolac, ketorolac tromethamine and droxicam.

**Table 8.** Summary of Major GI Events with Piroxicam-β-cyclodextrin, Piroxicam and Other Reference Agents in 28 Chronic Treatment Studies From a Pooled Safety Analysis (Data On File, Chiesi Farmaceutici)

	Piroxicam-β-cyclodextrin^a^		Piroxicam		Other^b^	
	N=15 040		N=579		N=314	
	N	%	N	%	N	%
Black feces	0	0.00	0	0.00	0	0.00
Bleeding	14	0.09	0	0.00	2	0.64
Erosions	0	0.00	0	0.00	0	0.00
Melena	11	0.07	4	0.69	0.	0.00
Perforations	0	0.00	0	0.00	0	0.00
Rectal hemorrhage	1	0.01	6	1.04	0.	0.00
Ulcers (duodenal)	1	0.01	6	1.04	0	0.00
Ulcers (peptic)	3	0.02	0	0.00	2	0.64
Ulcers (stomatitis)	0	0.00	0	0.00	1	0.32
Ulcus duodeni	3	0.02	0	0.00	0	0.00
Ulcus ventriculi	2	0.01	0	0.00	0	0.00

a Includes all dosage forms and all doses of PBC

b Other reference agents included tenoxicam, diclofenac/diclofenac sodium, meclofenamate sodium, nabumetone, etodolac, ketorolac tromethamine and droxicam

## Footnotes

1 The term “coxib” is being used in the medical/scientific literature as synonymous of “selective COX-2 inhibitor”, which is not the case. The term “coxib” is a World Health Organization term used to describe a *chemical* class. It does not describe any pharmacological activity, nor indicate anything regarding COX-2 selectivity. Actually, there are members of the coxib family (e.g. SC-560) that selectively inhibit COX-1 [12].

2 Actually, in order to increase their solubility, chemically modified CDs have been synthesized, of which the hydroxypropyl-cyclodextrin derivatives (HP-CDs), with low degree of substitution, showed the best complexing properties [77].

3 Supercritical carbon dioxide is a fluid state of carbon dioxide where it is held at or above its critical temperature and critical pressure. Carbon dioxide usually behaves as a gas in air at standard temperature and pressure (STP), or as a solid, designated as dry ice when frozen. If the temperature and pressure are both increased from STP to be at or above the critical point for carbon dioxide, it can adopt properties midway between a gas and a liquid. More specifically, it behaves as a supercritical fluid above its critical temperature (31.1 °C) and critical pressure (72.9 atm/7.39 MPa), expanding to fill its container like a gas but with a density like that of a liquid. Supercritical CO_2_ is a peculiar solvent due to its low toxicity and environmental impact. The relatively low temperature of the process and the stability of CO_2_ also allows most compounds to be extracted with little damage or denaturing.
